# Calcium-dependent activator protein for secretion 2 is involved in dopamine release in mouse midbrain neurons

**DOI:** 10.3389/fnmol.2024.1444629

**Published:** 2024-07-18

**Authors:** Hirotoshi Iguchi, Takumi Katsuzawa, Chihiro Saruta, Tetsushi Sadakata, Shota Kobayashi, Yumi Sato, Akira Sato, Yoshitake Sano, So Maezawa, Yo Shinoda, Teiichi Furuichi

**Affiliations:** ^1^Department of Applied Biological Science, Faculty of Science and Technology, Tokyo University of Science, Noda, Japan; ^2^Department of Environmental Health, School of Pharmacy, Tokyo University of Pharmacy and Life Sciences, Hachioji, Japan; ^3^Laboratory for Molecular Neurogenesis, RIKEN Brain Science Institute, Wako, Japan; ^4^Education and Research Support Center, Gunma University Graduate School of Medicine, Maebashi, Japan; ^5^Department of Basic Pathology, Fukushima Medical University School of Medicine, Fukushima, Japan; ^6^Laboratory for Molecular Mechanisms of Brain Development, Center for Brain Science, RIKEN, Wako, Japan

**Keywords:** CADPS2, dopamine, exocytosis, midbrain, tyrosine hydroxylase

## Abstract

The Ca^2+^-dependent activator protein for secretion (CAPS/CADPS) family protein facilitates catecholamine release through the dense-core vesicle exocytosis in model neuroendocrine cell lines. However, it remains unclear if it induces dopamine release in the central neurons. This study aimed to examine the expression and function of CADPS2, one of the two CADPS paralogs, in dopamine neurons of the mouse midbrain. This study shows that CADPS2 was expressed in tyrosine hydroxylase and the vesicular monoamine transporter 2 (VMAT2)-positive dopaminergic neurons of the midbrain samples and primary mesencephalic cell cultures. Subcellular fractions rich in dopamine were collected using immunoaffinity for CADPS2 from midbrain protein extracts. Cell imaging using fluorescent false neurotransmitter FFN511 as a substrate for VMAT2 showed decreased activity-dependent dopamine release in *Cadps2*-deficient cultures, compared to that in wild-type cultures. These results suggest that CADPS2 is involved in dopamine release from the central neurons, indicating its involvement in the central dopamine pathway.

## 1 Introduction

The Ca^2+^-dependent activator protein for secretion (CAPS/CADPS) regulates the activity-dependent exocytosis of dense-core vesicles (DCVs) (Walent et al., 1992; Grishanin et al., 2004). CAPS2/CADPS2 is one of two CADPS family members in mammals (Speidel et al., 2003; Sadakata et al., 2004). We previously showed that CADPS2 is localized in tyrosine hydroxylase (TH)-positive dopamine (DA) neurons in the mouse ventral tegmental area (VTA) and substantia nigra pars compacta (SNc) (Sadakata et al., 2006). A possible link between CADPS2 and central dopamine (DA) function is also suggested by the fact that a maternally inherited variation of *CADPS2* gene in patients with autism spectrum disorder disrupts the interaction of CADPS2 with the DA receptor type 2 (Bonora et al., 2014) and by the discovery of a small neuronal cell cluster characterized by *CADPS2* overexpression and low TH levels in the midbrains of patients with idiopathic Parkinson’s disease (Smajić et al., 2022).

The CADPS protein family is involved in the release of catecholamine from PC12 cells (Grishanin et al., 2002; Speidel et al., 2003), a model neuroendocrine cell line; however, it remains unclear whether CADPS proteins are involved in the release of central DA neurons in the midbrain.

Therefore, to elucidate the role of CADPS2 in central DA neurons, we aimed to analyze the cellular expression of *Cadps2* mRNA and CADPS2 protein in TH-positive neurons of the SNc and VTA, the association of CADPS2 protein with DA-rich subcellular fractions of midbrain extracts, and the DA release in primary midbrain neuron cultures prepared from *Cadps2* knockout (KO) and wild-type (WT) mice.

## 2 Methods

### 2.1 Animals

C57BL/6 J and ICR mice were purchased from the Nihon SLC (Hamamatsu, Japan). *Cadps2* KO mice (C57BL/6-*Cadps2^tm1Tfr^*) (Sadakata et al., 2007) were used. The mice were housed in an environment with a 12-h/12-h light/dark cycle (daytime 8:00–20:00) with controlled temperature (23 ± 2°C) and humidity (55 ± 10%) and *ad libitum* access to food and water. All animal studies were conducted following the recommendations and protocols approved by the Animal Care and Use Committee of the Tokyo University of Science (approval number: N16008, N17003, N18003, N19003) and international guidelines.

### 2.2 Antibodies and immuno-histochemical and -cytochemical analyses

Guinea pig and rabbit polyclonal anti-CADPS2 antibodies (1 ng/μL) were used as described previously (Sadakata et al., 2004, 2006). We also used mouse monoclonal anti-TH (1:100; MAB318, RRID: AB_2201528, Merck-Millipore, Darmstadt, Germany), rabbit polyclonal anti-vesicular monoamine transporter 2 (VMAT2) (1/500; ab81855, RRID: AB_2188123, Abcam plc, Cambridge, United Kingdom), rabbit polyclonal anti-calbindin D-28 k (1:2,000; AB1778, RRID: AB_2068336, Merck-Millipore), and chicken polyclonal anti-microtubule associated protein 2 (MAP2) (1:2,000; AB5543, RRID: AB_571049, Merck-Millipore) antibodies. As fluorescent secondary antibodies (Invitrogen-ThermoFisher Scientific, Waltham, MA, United States), we used Alexa Fluor 488 goat anti-guinea pig IgG (H + L) (1/3,000; A11073, RRID: 2534117), Alexa Fluor 555 donkey anti-mouse IgG (H + L) (1/3,000; A21202, RRID: AB_141607), Alexa Fluor 647 goat anti-rabbit IgG (H + L) (1/3,000; A21244, RRID: AB_2535812), and Alexa Fluor 647 goat anti-chicken IgG (1/3,000, A21449, RRID: AB_2535866) antibodies. Immunohistochemistry (IHC) and immunocytochemistry analyses were performed as described previously (Sadakata et al., 2004, 2006). The immunostained samples were imaged using an epifluorescent microscope (Eclipse E800; Nikon, Tokyo, Japan), equipped with a cooled CCD camera (SPOT model 1.3.0; Diagnostic Instruments) or with a confocal laser microscope (FV1000 IX81; Olympus), fitted with oil immersion objective lenses UPlanSApo 100× (NA 1.40, resolution 0.168 μm/pixel) and PLAPON 60× (NA 1.42, resolution 0.103 μm/pixel), and an objective lens UPLSAPO 20× (NA0.75, resolution 0.31 μm/pixel). Images were acquired using NIS-elements (Nikon) and FV-10 ASW software (OLYMPUS) and analyzed using the ImageJ software.

### 2.3 Primary mesencephalic cell cultures

Primary mesencephalic cell cultures on an astrocyte feeder layer were prepared as previously described (Fasano et al., 2008; Onoa et al., 2010) with several modifications. Mouse astrocyte monolayers were prepared from the cerebral cortex of C57BL/6 J at P0–P3. The midbrain regions were dissected at embryonic days 15–17. Dissociated cells suspended in Neurobasal A + (Neurobasal A medium [10888-022, Gibco, ThermoFisher Scientific, MA, United States], 10% FBS, 2% B-27 Supplement [17504, Gibco], 1% GlutaMax [35050, Gibco], and 1% Penicillin–Streptomycin) at a cell density of 1.0 × 10^5^ cells/mL were plated on an astrocyte feeder layer in the presence of 10 ng/mL GDNF (079-0611, FUJIFILM Wako, Osaka, Japan). Notably, 24–48 h after the start of incubation, 5-fluorodeoxyuridine (BD5199B, Gibco) and uridine (U6381, Gibco) were added to the culture to restrict astrocyte growth (Onoa et al., 2010).

### 2.4 Time-lapse FNN511 fluorescent cell imaging

Cultured cells (DIV12) were incubated in a loading solution containing 12.5 nM FFN511 trifluoroacetate salt hydrate (F9932-5MG, Sigma-Aldrich, St. Louis, MO, United States) in HBSS at 37°C for 10–15 min. FFN511-loaded cells were rinsed thrice in a low potassium (K^+^) solution (150 mM NaCl, 4 mM KCl, 2 mM MgCl_2_, 2 mM CaCl_2_, 10 mM glucose, 10 mM HEPES, pH 7.3, and 310 Osmol), and then used for fluorescent live cell imaging. FFN511-loaded cells on a coverslip were placed in a low K^+^ solution and analyzed for 10-min under fluorescent imaging at room temperature. The cells were depolarized 30 s after the start of the imaging by adding a high K^+^ solution (58 mM NaCl, 96 mM KCl, 2 mM MgCl_2_, 2 mM CaCl_2_, 10 mM glucose, 10 mM HEPES, pH 7.3, and 310 Osmol) in half volume (final concentration of K^+^ = 50 mM). Changes in FFN511 fluorescence were monitored using a fluorescent microscope Nikon ECLIPSE Ti-U (Nikon, with 436AF20 excitation filter, 455DRLP dichroic mirror, and 480AF49 emission filter) equipped with an Apo TIRF 100× oil DIC N2 objective (NA 1.49, resolution 0.16 μm/pixel) and an EM-CCD camera (ANDOR Technology, iXON DU-897, Belfast, Northern Ireland). Images were acquired with 30 ms exposure time, multiplier 300, binning 1 × 1 using a NIS-elements software (Nikon) and analyzed using the ImageJ software.

### 2.5 Dopamine assay of immunoaffinity-purified midbrain subcellular fractions

For each experiment, the midbrains of 10 mice were homogenized in 5 mL of ice-cold 50 mM HEPES (pH 7.4), 5 mM EDTA, 0.32 M sucrose and a protease inhibitor mixture (cOmplete, Mini, EDTA free, 11836170001, Sigma-Aldrich) with a glass-Teflon homogenizer followed by centrifugation at 800×*g* for 10 min at 4°C. The resultant supernatant was mixed with 9 vol. of ice-cold hypotonic solution (Milli-Q water). Immuno-magnet beads (Dynabeads, M-280, Dynal, Lake Success, NY, United States) bound to rabbit anti-CADPS2 antibody or control rabbit IgG were prepared and used for immunoaffinity selection as previously described (Sadakata et al., 2004). The immunoaffinity-purified fractions were eluted by incubation in 100 μL of 0.01 N HCl. They were subjected to DA enzyme immunoassay (EIA) using the DA EIA kit (Dopamine Research EIA, BA 10–5,300, Labor Diagnostika Nord GmbH & Co.KG, Nordhorn, Germany) following the manufacturer’s protocol (sensitivity: 8.3 pg/100 μL). DA concentrations in vesicles were determined at 450 nm of the absorbance based on known standards, and the experiments were performed in triplicate (*n* = 3).

### 2.6 Statistical analysis

The number of animals used in each experimental setting (*n*) and the analysis performed are specified in the figure legends. An investigator blinded to the genotypes of the mice from which the tissues were derived performed the evaluation. Data were analyzed using the Student *t*-test, except for the analysis of DA release rates (two-way ANOVA) and the subgroup analysis of DA release levels (two-way ANOVA with post-hoc Turkey-Kramer test). Values are presented as the mean ± standard error of mean. Approximate straight lines (slopes) were calculated using Microsoft Excel.

## 3 Results

### 3.1 CADPS2 is expressed in TH and VMAT2-expressing dopaminergic neurons and associated with dopamine-rich subcellular fractions in the midbrain

**Figure 1:**
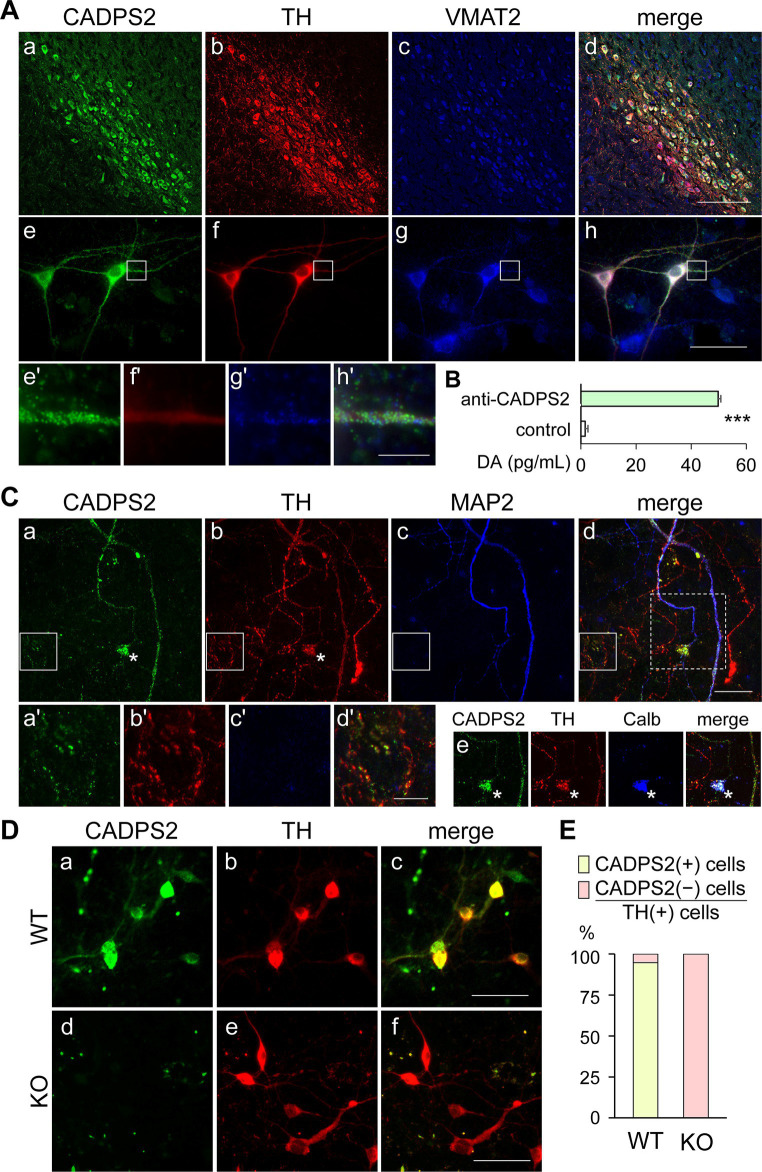
CADPS2 protein localizes to TH and VMAT2-positive neurons and is associated with DA-rich subcellular fractions of the midbrain, but is absent in the primary DA neurons of *Cadps2* KO mice. **(A)** Triple immunostaining for CADPS2 **(a,e)**, TH **(b,f)**, and VMAT2 **(c,g)** in the mouse SNc region **(a–d)** and midbrain primary cell cultures (DIV8) **(e–h)**. Panels **(d,h)** are merged images presented in **(a–c)** and **(e–g)**, respectively. Images **(e’–h’)** represent magnified views of white squares in panels **(e–h)**, respectively. Scale bars in **d**, **h**, and **h’** = 100, 50, and 25 μm, respectively. **(B)** DA EIA showing DA concentration (pg/mL) in the subcellular fractions immunoaffinity-purified using control IgG or anti-CADPS2 antibody. Three experiments (*n* = 3) were performed using 10 mouse midbrains in each experiment. DA contents (mean ± SEM): control IgG 1.53 ± 2.15 (*n* = 3); anti-CADPS2 antibody 16.60 ± 0.23 (*n* = 3). ***p = 0.0022. **(C)** Triple immunostaining for CADPS2 **(a)**, TH **(b)**, and MAP2 **(c)** in primary mesencephalic cell cultures (DIV14). Panel **(d)** represents a merged image. Images **(a’–d’)** represent magnified views of the white squares in panels **(a–d)**, respectively. The four composite images in panel **(e)** are cropped images [corresponding to the square enclosed by the white dotted line in panel **(d)**] from panels **(a–d)**, respectively, except that MAP2 **(c)** and merged image **(d)** have been replaced with calbindin (Calb) and CADPS2-TH-Calb immunosignals, respectively. Asterisks in **(a,b,e)** represent Calb-positive cell bodies likely corresponding to VTA DA neurons. Scale bars in **d** and **d’** = 50 and 25 μm, respectively. **(D)** Double immunostaining for CADPS2 **(a,d**) and TH **(b,e)** in primary mesencephalic cell cultures (DIV7) of WT **(a–c)** and *Cadps2* KO **(d–f)** mice. Panels **(c,f)** represent merged images. Scale bar, 50 μm. **(E)** Cell ratio (%) of immunoreactivity to CADSPS2 and TH in five independent midbrain cell cultures obtained from WT and KO mice. The ratio of CADPS2-positive (+) vs. negative (−) cells in TH-positive (+) cells: 94.7% in WT [*n* = 57; 54 (+) cells and 3(−) cells]; 0% in KO [*n* = 88; 0 (+) cells and 88 (−) cells].

We first verified *Cadps2* mRNA expression in TH-positive DA neurons by double staining for *Cadps2* mRNA and TH protein using ISH and IHC, respectively. *Cadps2* mRNA signals were mainly localized in the cell bodies of TH-positive cells in the VTA and SNc (Supplementary Figures S1A,B), indicating that the *Cadps2* gene is expressed in DA neurons of the SNc and VTA. This finding is consistent with those showing that CADPS2 protein is expressed in TH-positive neurons in both the SNc and VTA (Supplementary Figure S1C). Next, we confirmed that CADPS2 immunosignals were detected in TH-positive DA neurons co-expressing VMAT2 (which is responsible for packing DA into secretory vesicles) in mouse midbrain regions (Figures 1Aa–d) and in the primary midbrain cultured cells (Figures 1Ae–h). Although there were slight differences in the immunosignal patterns and intensities of the three molecules, they were generally co-expressed in the cell bodies and proximal neurites (Figures 1Ae’–h’).

To assess the possible association between CADPS2 proteins and DA-containing vesicles, subcellular fractions prepared from midbrain extracts were subjected to the immunoaffinity selection using anti-CADPS2 antibody- or control IgG-bound magnet beads, followed by DA EIA. Therefore, the subcellular fraction associated with CADPS2 exhibited higher DA content than that treated with control IgG beads (Figure 1B).

We analyzed the expression patterns of CADPS2 in neurites of cultured DA neurons. Strong CADPS2 immunosignals were localized in TH and MAP2-positive dendrites (Figures 1Ca–d) of SNc and calbindin-positive VTA neurons (Figure 1Ce). There were some TH-positive neurites with very weak or punctate immunosignals for CADPS2, which may reflect altered expression of these molecules in the midbrain (Figure 1A). CADPS2 signals were detected in some TH-positive and MAP2-negative neurites (Figures 1Ca’–d’), suggesting that CADPS2 is localized in neurites other than the dendrites, likely the axons.

### 3.2 Dopaminergic neurons in *Cadps2*-deficient mice express TH but not CADPS2

We compared the expression of CADPS2 proteins in primary midbrain cell cultures between *Cadps2* KO and WT mice. Approximately 95% of TH-positive cells were detectable to express CADPS2 proteins in WT cultures (Figures 1D,E). However, no TH- and CADPS2-double-positive cells were observed in the *Cadps2* KO cultures tested (Figures 1D,E). Therefore, we assumed that the primary cultures of two genotypes could be used to compare DA release in the presence and absence of CADPS2 function.

### 3.3 CADPS2 deficiency causes an impairment in dopamine release from primary mesencephalic cell cultures

**Figure 2:**
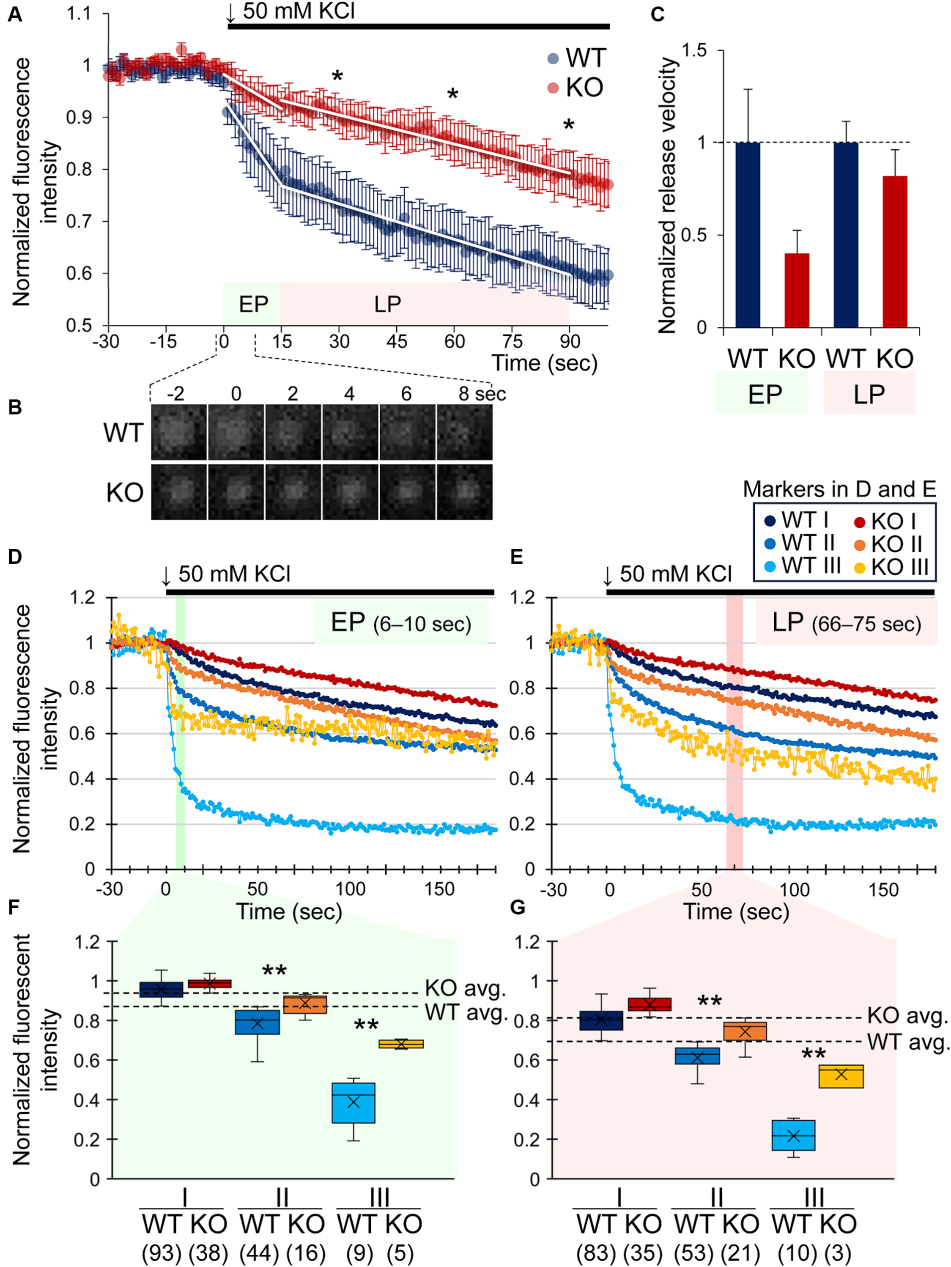
Early and late phases of DA release are attenuated by CADPS2 deficiency in dopaminergic neuron cultures. **(A)** Changes in normalized intensities of FNN511 fluorescent puncta (*ΔF/F_0_* = 1) recorded from mostly somatodendritic areas of 10 and 8 independent cultures (coverslips) from WT (*n* = 10) and *Cadps2* KO (*n* = 8) mice, respectively, upon depolarization with 50 mM KCl at time 0. *White lines* represent the temporal changes shown as the slope of the plot that was conveniently divided into two phases, “early phase (EP)” between 0 and 15 s, WT: *y* = − 0.0109 *x* + 1.2628 and KO: *y* = − 0.0044 *x* + 1.1146; “late phase (LP)” between 15 and 90 s, WT: *y* = − 0.0023 *x* + 0.8706 and KO: *y* = − 0.0019 *x* + 1.0168. *p* values were calculated at – 15, 0, 15, 30, 60, and 90 s, and the latter three time points of the LP showed statistically significant effects: *p < 0.05. **(B)** Consecutive images of a representative FFN511 fluorescent punctum in WT and KO cultures between −2 and 8 s. **(C)** Normalized release velocity of FFN511 puncta in KO cultures to those in WT cultures. The normalized release velocity was calculated from the slope of the fitted curve in **(A)**. The slope of the KO showed approximately 40.4 and 82.6% of those of the WT in the EP and LP, respectively. Two-way ANOVA *p* = 0.091 and 0.005 for WT vs. KO and EP vs. LP, respectively (WT *n* = 10, KO *n* = 8). **(D–G)** Changes in normalized intensity of fluorescent puncta in three subgroups based on the fluorescence intensity from 6 to 10 s (EP) **(D,F)** and 66 to 75 s (LP) **(E,G)**. Puncta shown in **(A)** were subdivided into three subgroups, I, II, and III, against the average value (avg.) within defined time windows in the EP and LP, respectively [shown by *dashed lines* in **(F,G)**]. EP (time window: 6–10 s) WT avg. = 0.870, KO avg. = 0.933; LP (time window: 66–75 s) WT avg. = 0.694, KO avg. = 0.813. Major subgroups I (higher than the avg.) and II (lower than the avg.) and a minor subgroup III (lower than the avg. −2 × standard deviation) (Supplementary Table S1 and Supplementary Figure S2). In **(F,G)**, the whiskers (vertical lines), the line within the box, and × represent the minimum and maximum values, the median, and the mean, respectively. Numbers in parentheses on the bottom of the horizontal axis indicate the number of puncta in each subgroup. *p* values: *, <0.05, **, <0.01.

To examine if CADPS2 is involved in DA release in central neurons, we compared activity-dependent DA release from primary mesencephalic cell cultures of WT and *Cadps2* KO mice using a fluorescent false neurotransmitter FFN511, a VMAT2 substrate. FFN511-loaded cultures were chemically induced for depolarization, and fluorescent puncta images were acquired near the cell body and proximal neurites. From the overall pattern, the curves of fluorescence intensity changes could be evaluated using two components of approximate straight lines (slopes), the “early phase (EP)” at 0–15 s and the subsequent “late phase (LP)” at 15–90 s (Figures 2A,B). The changes in fluorescence intensity were attenuated in KO cultures than in WT cultures in the LP at 30, 60, and 90 s (Figure 2A). The transient time series from EP to LP showed significant variation (*p* = 0.005) with no interaction between genotype and velocity (Figure 2C).

There was variability in the release capacity among FFN511 puncta observed in WT and KO cultures, most especially in the EP, which was probably influenced by the properties of vesicle pools before release induction. To simplify the analysis, we re-plotted the change in fluorescence intensity after depolarization by focusing on typical time widows, 5 s of the EP (at 6–10 s) and 10 s of the LP (at 66–75 s), and subdividing the fluorescent puncta with the heterogeneous intensity into three subgroups (two major subgroups I and II, and a minor subgroup III) based on the average value (avg.): I (higher intensity than the average), II (lower than the average) and III (considerably low intensity) (Figures 2D–G) (Supplementary Table S1). The subgroup III puncta were detected in specific cell regions (Supplementary Figure S2). As a result, fluorescence changes in KO cultures were significantly attenuated compared with WT cultures in the major subgroup II and the minor subgroup III of the EP (Figures 2D,F) and LP (Figures 2E,G), except for subgroup I, which showed slight changes in fluorescence intensity, suggesting that the loss of CADPS2 in KO cultures affects the early and late phases of more active DA release after induction.

## 4 Discussion

A regulatory role of CADPS family proteins in the release of catecholamine has been reported mainly in neuroendocrine cells (Berwin et al., 1998; Speidel et al., 2003). In this study, we showed that CADPS2 is critical, but not essential, to the efficient DA release from central DA neurons. CADPS2 was localized near and/or around TH-positive sites in the mouse midbrain SNc and VTA and was co-expressed with TH and VMAT2 in primary mesencephalic cell cultures. In addition, the CADPS2-associated subcellular fractions prepared from midbrain extracts exhibited high DA contents. We also revealed that the primary mesencephalic cell cultures of *Cadps2* KO mice have a lower DA release compared with those of WT cultures. Therefore, our results demonstrate that CADPS2 is involved in the DA release in mouse brains.

There appears to be some heterogeneity in the DA release events imaged in the present study, possibly due to different properties of the synaptic vs. somatodendritic DA vesicles filled by VMAT2 (Nirenberg et al., 1996). These differences may include DCVs or SVs, FFN511-uptake ability, local vesicle density, readily releasable or reserve pools, partial or full release, and the difference in release mechanisms (e.g., involvement of different synaptotagmin subtypes) (Delignat-Lavaud et al., 2022, 2023; Lebowitz et al., 2023) and kinetics from the somatodendrites or axon terminals and those from SNc or VTA neurons (Rice and Patel, 2015). CADPS family proteins regulate the priming step of exocytosis, thereby affecting the readily releasable pool size of DCVs (for catecholamine and neuropeptide release) or SVs (for glutamate release) (Speidel et al., 2003; Grishanin et al., 2004; Shinoda et al., 2011, 2016). Based on these perspectives, we suggest that CADPS2 deficiency impairs DA release from the immediate/readily and resting releasable pools (probably corresponding to the EP and LP, respectively, in this study).

We monitored DA release mostly around the soma and proximal neurites of cultured DA neurons. FFN511 can be loaded into SVs and DCVs through the VMAT2. Therefore, in the future, we aim to investigate whether CADPS2 exerts its role on SV or DCV exocytosis in axons or somatodendrites of VTA or SNc DA neurons and analyze whether any phenotypes exhibited by *Cadps2* KO mice (Sadakata et al., 2007, 2012) are associated with DA-related diseases.

## Data availability statement

The datasets presented in this study can be found in online repositories. The names of the repository/repositories and accession number(s) can be found in the article/Supplementary material.

## Ethics statement

The animal study was approved by Animal Care and Use Committee of the Tokyo University of Science. The study was conducted in accordance with the local legislation and institutional requirements.

## Author contributions

HI: Conceptualization, Investigation, Methodology, Writing – original draft, Writing – review & editing. TK: Data curation, Writing – review & editing. CS: Writing – review & editing, Investigation, Methodology. TS: Investigation, Methodology, Writing – review & editing. SK: Investigation, Methodology, Writing – review & editing. YS: Data curation, Investigation, Methodology, Writing – review & editing. AS: Data curation, Writing – review & editing. YSa: Data curation, Writing – review & editing. SM: Data curation, Writing – review & editing. YSh: Data curation, Funding acquisition, Supervision, Writing – review & editing. TF: Conceptualization, Funding acquisition, Supervision, Writing – original draft, Writing – review & editing.
