# Supplementary material for: Calcium-dependent activator protein for secretion 2 is involved in dopamine release in mouse midbrain neurons
Source: Front Mol Neurosci. 2024 Jul 18;17:1444629. doi: 10.3389/fnmol.2024.1444629 (PMC11291307; doi:10.3389/fnmol.2024.1444629)
Supplement: Supplementary file 1 [file Data_Sheet_1.PDF]

Supplementary Material

CAPS2/CADPS2 is involved in dopamine release in mouse midbrain neurons

Hirotoshi Iguchi[1], Takumi Katsuzawa[2], Chihiro Saruta[3], Tetsushi Sadakata[3, 4], Shota Kobayashi[1], Yumi Sato[3, 5], Akira Sato[1, 3], Yoshitake Sano[1], Sho Maezawa[1], Yo Shinoda[2, 3, *], and Teiichi Furuichi[1, 3, 6, *]

[1]Department of Applied Biological Science, Faculty of Science and Technology, Tokyo University of Science, Noda, Japan

[2]Department of Environmental Health, School of Pharmacy, Tokyo University of Pharmacy and Life Sciences, Hachioji, Japan

[3]Laboratory for Molecular Neurogenesis, RIKEN Brain Science Institute, Wako, Japan

[4]Education and Research Support Center, Gunma University Graduate School of Medicine, Maebashi, Japan

[5]Department of Basic Pathology, Fukushima Medical University School of Medicine, Fukushima, Japan

[6]Laboratory for Molecular Mechanisms of Brain Development, Center for Brain Science, RIKEN, Wako, Japan

*** Correspondence:**
Teiichi Furuichi
tfuruichi@rs.tus.ac.jp

Yo Shinoda
shinoda@toyaku.ac.jp

1 Supplementary Methods

1.1 *In situ* hybridization

In situ hybridization (ISH) was conducted as described previously (Sadakata et al., 2004; Sadakata et al., 2006).The cDNA fragments, which were amplified with PCR using specific primer sets (for *Cadps2* forward primer [F] 5'-GAGAGCCGCGAGGTGTTGGT-3' and reverse primer [R] 5'-CTGCAGGCGGATGCGCCGCT-3', 216 bp; for *TH* F 5'-GAAAGTGTTTGAGACATTTGAA-3' and R 5'-CAATTTCCTCCTTTGTGTATTC-3', 389 bp), were used as template sequences to generate riboprobes labeled with digoxigenin-11-UTP (11209256910, Roche Diagnostics GmbH, Mannheim, Germany). Hybridization signals were detected using a digoxigenin detection kit (11175041910, Roche). ISH sections were subjected to the sequential reaction of IHC with an anti-TH antibody (1:100) and the biotin-conjugated anti-mouse IgG (1:1,000), followed by immunodetection using a VECTASTAIN ABC-HRP kit (PK-4000, VECTOR Laboratories, Newark, CA, USA) and 0.05% 3,3'-diaminobenzidine/0.01% H_2O_2.

Sadakata, T., Itakura, M., Kozaki, S., Sekine, Y., Takahashi, M., and Furuichi, T. (2006). Differential distributions of the Ca2+ -dependent activator protein for secretion family proteins (CAPS2 and CAPS1) in the mouse brain. J. Comp. Neurol. 495, 735-753. doi: 10.1002/cne.20947

Sadakata, T., Mizoguchi, A., Sato, Y., Katoh-Semba, R., Fukuda, M., Mikoshiba, K., et al. (2004). The secretory granule-associated protein CAPS2 regulates neurotrophin release and cell survival. J. Neurosci. 24, 43-52. doi: 10.1523/jneurosci.2528-03.2004

2 Supplementary Figure

2.1 Supplementary Figure 1

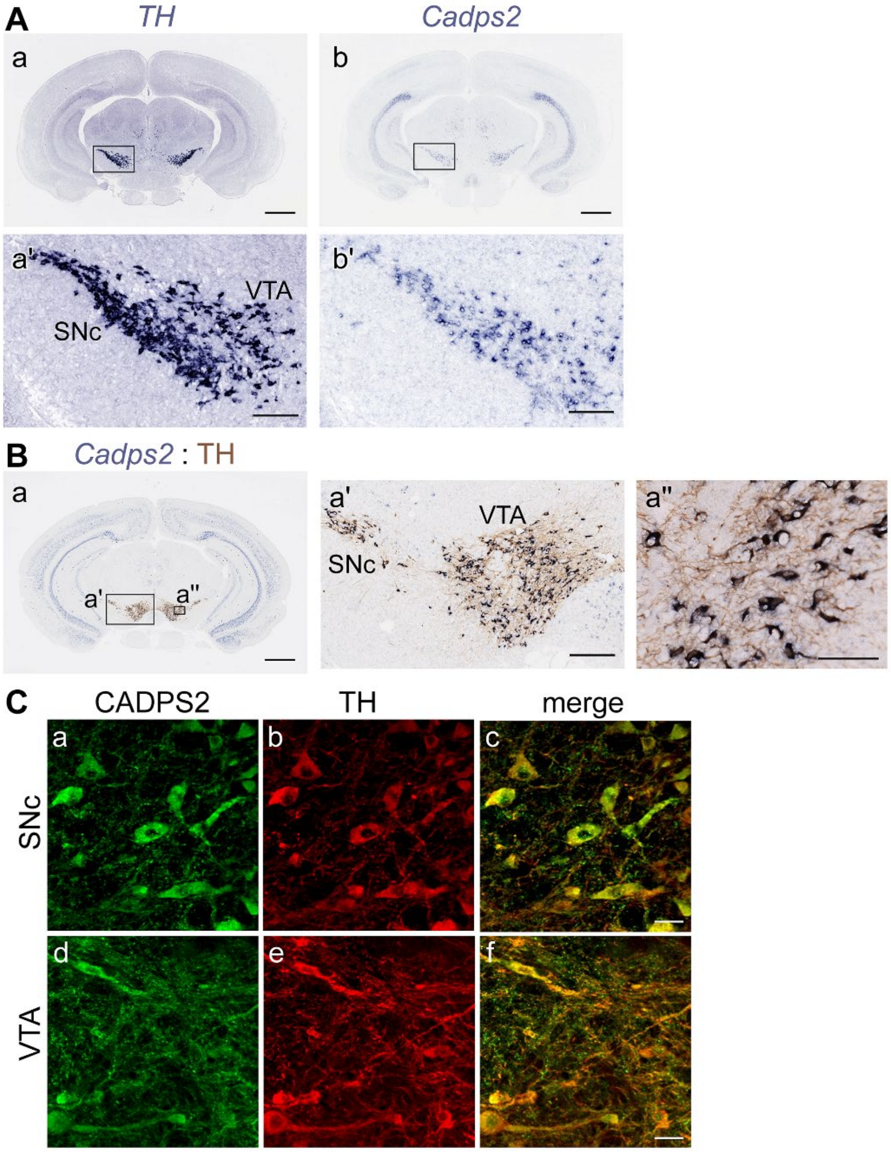

Supplementary Figure 1. Expression of CADPS2 mRNA in dopaminergic neurons of the mouse ventral midbrain

(**A**) *In situ* hybridization (ISH) staining for tyrosine hydroxylase (*TH*) (**a**) and *Cadps2* (**b**) mRNA levels in adult mouse brains were derived from five coronal or sagittal sections obtained from four mice. **a'** and **b'** represent magnified images of the SNc-VTA area indicated by rectangles in **a** and **b**, respectively. Scale bars, 1 cm in panels **a** and **b** and 200 µm in panels **a'** and **b'**. (**B**) (**a**) Double staining for *Cadps2* mRNA (ISH, *blue/purple* color) and TH protein (IHC, *brown color*) were derived from three coronal or sagittal sections obtained from three mice. The panels **a'** and **a''** represent magnified images indicated by rectangles marked **a'** and **a''** in panel **a**, respectively. Scale bars, 1 cm, 200 µm, and 40 µm in panels **a**, **a'** and **a''**, respectively. (**C**) Double immunostaining for CADPS2 (**a**, **d**) and tyrosine hydroxylase (TH) (**b**, **e**) in the SNc (**a**–**c**) and VTA (**d**–**f**) of mice. Panels **c** and **f** are merged images in SNc and VTA, respectively. Scale bar, 20 µm.

2.2 Supplementary Figure 2

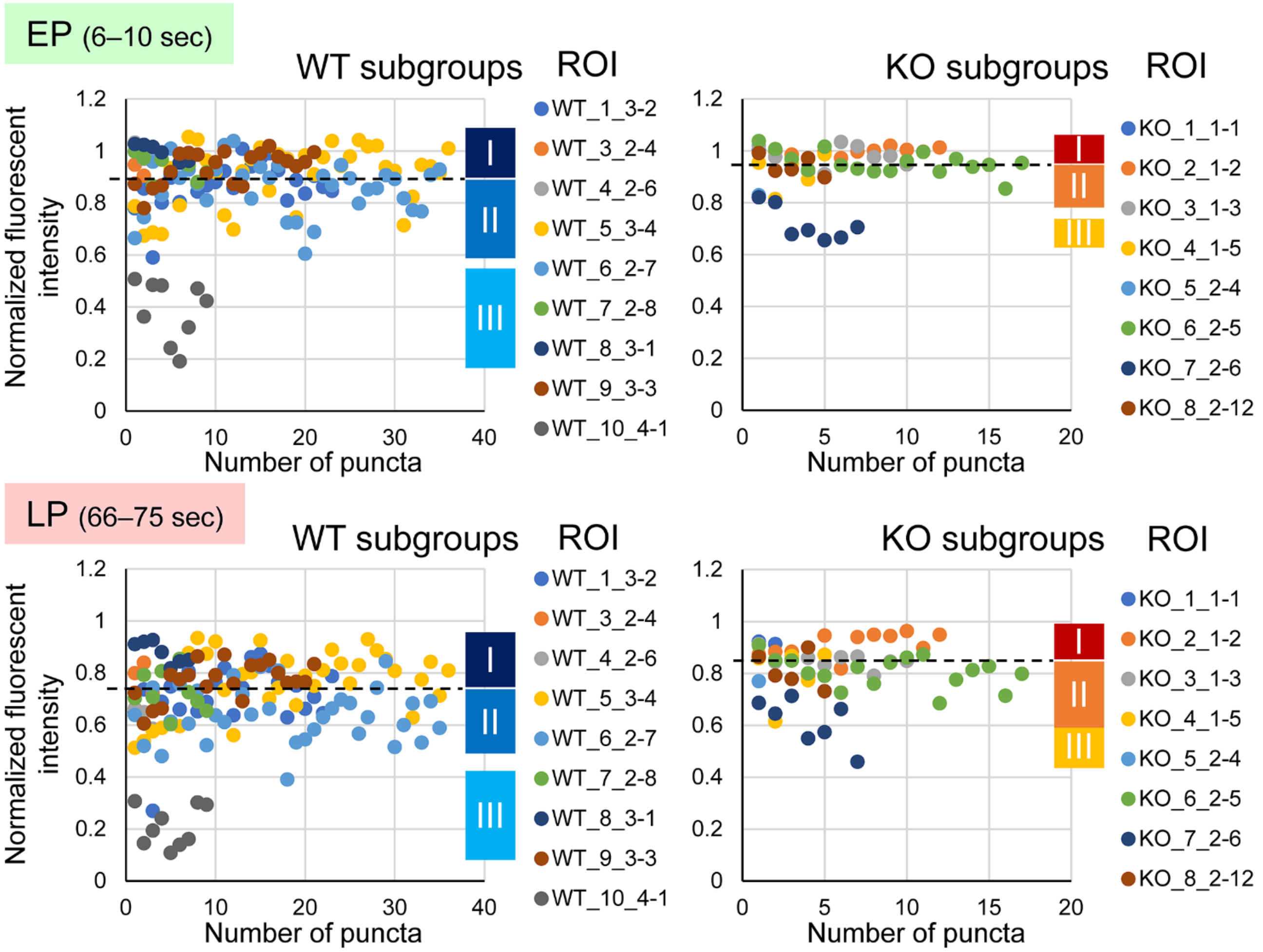

Supplementary Figure 2. Three subgroups (I–III) of FFN511 puncta arbitrarily subdivided in relation to the average line of total fluorescence intensity changed in the early and late release phases (EP and LP) in cultured dopaminergic neurons of *Cadps2* KO and WT mice.

Boxes I–III on the right side of the plot indicate the subgroups, respectively. FFN511 fluorescent signals were obtained from ROIs in nine and eight independent cultures for WT and KO, respectively, each of which include different numbers of fluorescent puncta (*X*-axis) with different fluorescent intensities (*Y*-axis). The color of the markers (corresponding to puncta) and that of the ROI match; therefore, markers of the same color indicate that they were detected in the same area (ROI). Three subgroups are arbitrarily subdivided as follows: the fluorescence intensity values of subgroup I are higher than the total average value (Avg.) (*black dashed line*), those of subgroup II are lower than the Avg. and higher than the value of the Avg. – 2 x standard deviation (σ) (*red dashed line*), and those of subgroup III are lower than the value of the Avg. – 2 x σ. The values of the Avg. and Avg. – 2 x σ are described in Supplementary Table 1. Most of the puncta in subgroup III were measured in the ROIs of WT_10_4-1 and KO_7_2-6 and showed relatively larger changes compared with the other puncta.

3 Supplementary Table

3.1 Supplementary Table 1. FFN511 fluorescence intensity levels of the three subgroups (I–III) were subdivided in relation to the average total fluorescence intensity changed in the early release phase (EP) and late release phase (LP) in cultured dopaminergic neurons of *Cadps2* KO and WT mice.

Release phases (time)	Geno-types	Total	Major subgroup I (>Avg.)	Major subgroup II (Avg. −2σ < ∧ ≤ Avg.)	Minor subgroup III (≤ Avg. −2σ)
		Avg. (n)	Min – Max<br>Avg. ± SEM<br>(n)	Min – Max<br>Avg. ± SEM<br>(n)	Min – Max<br>Avg. ± SEM<br>(n)
EP (6–10 sec)	WT	0.870181 (146)	0.872694 – 1.054699<br>0.957248 ± 0.004827<br>(93)	0.590942 – 0.868665<br>0.784865 ± 0.011184<br>(44)	0.191295 – 0.507772<br>0.387587 ± 0.038356<br>(9)
	KO	0.933192 (59)	0.938225 – 1.037999<br>0.985469 ± 0.004296<br>(38)	0.801815 – 0.931333<br>0.888105 ± 0.011768<br>(16)	0.655617 – 0.705799<br>0.680164 ± 0.009112<br>(5)
LP (66–75 sec)	WT	0.693852 (146)	0.697529 – 0.934151<br>0.804079 ± 0.006798<br>(83)	0.390198 – 0.691292<br>0.611421 ± 0.008676<br>(53)	0.107916 – 0.306400<br>0.215853 ± 0.023814<br>(10)
	KO	0.813395 (59)	0.818355 – 0.962985<br>0.879991 ± 0.006836<br>(35)	0.614558 – 0.812758<br>0.743253 ± 0.012507<br>(21)	0.459325 – 0.573799<br>0.527432 ± 0.034788<br>(3)

FFN511 fluorescence intensities of three subgroups (two major subgroups, I and II, and a minor subgroup, III) at 6–10 s (within the EP) and 66–75 s (within the LP) after depolarization are indicated. The three subgroups of WT and KO cultures are arbitrarily subdivided as follows: the fluorescence intensity values of subgroup I are higher than the total average value (Avg.), those of subgroup II are lower than the Avg. and higher than the value of the Avg. – 2 x Standard deviation (σ), and those of subgroup III are lower than the value of the Avg. – 2 x σ. In the column of Total, Avg. represents the average FFN511 fluorescence intensity of total puncta analyzed, and (n) indicates the number of total fluorescent puncta analyzed. In the column of each subgroup, Min – Max indicates the minimum and maximum value of fluorescence intensity of FFN551 puncta, Avg. ± SEM shows the average ± SEM of fluorescence intensity, and (n) indicates the number of fluorescence puncta. The values of the Avg. – 2 x σ: in the EP, WT = 0.553378 and KO = 0.745913; in the LP, WT = 0.353569 and KO = 0.606326.
